# Concentration and Duration of Indoxyl Sulfate Exposure Affects Osteoclastogenesis by Regulating NFATc1 via Aryl Hydrocarbon Receptor

**DOI:** 10.3390/ijms21103486

**Published:** 2020-05-15

**Authors:** Wen-Chih Liu, Jia-Fwu Shyu, Paik Seong Lim, Te-Chao Fang, Chien-Lin Lu, Cai-Mei Zheng, Yi-Chou Hou, Chia-Chao Wu, Yuh-Feng Lin, Kuo-Cheng Lu

**Affiliations:** 1 Graduate Institute of Clinical Medicine, College of Medicine, Taipei Medical University, Taipei 110, Taiwan; wayneliu55@gmail.com (W.-C.L.); 11044@s.tmu.edu.tw (C.-M.Z.); athletics910@gmail.com (Y.-C.H.); 2 Division of Nephrology, Department of Internal Medicine, Tungs’ Taichung MetroHarbor Hospital, Taichung City 435, Taiwan; jamespslim@gmail.com; 3 Division of Nephrology, Department of Internal Medicine, Taipei Medical University Hospital, Taipei Medical University, Taipei 110, Taiwan; fangtechao@gmail.com; 4 Department of Biology and Anatomy, National Defense Medical Center, Taipei 114, Taiwan; shyujeff@mail.ndmctsgh.edu.tw; 5 Division of Nephrology, Department of Internal Medicine, School of Medicine, College of Medicine, Taipei Medical University, Taipei 110, Taiwan; 6 TMU Research Center of Urology and Kidney, Taipei 110, Taiwan; 7 Division of Nephrology, Department of Medicine, Fu Jen Catholic University Hospital, School of Medicine, Fu Jen Catholic University, New Taipei City 242, Taiwan; janlin0123@gmail.com; 8 Division of Nephrology, Department of Internal Medicine, Shuang Ho Hospital, Taipei Medical University, New Taipei City 235, Taiwan; 9 Division of Nephrology, Department of Medicine, Cardinal Tien Hospital, School of Medicine, Fu Jen Catholic University, New Taipei City 231, Taiwan; 10 Division of Nephrology, Department of Medicine, Tri-Service General Hospital, National Defense Medical Center, Taipei 114, Taiwan; wucc@mail.ndmctsgh.edu.tw; 11 Division of Nephrology, Taipei Tzu Chi Hospital, Buddhist Tzu Chi Medical Foundation, and School of Medicine, Buddhist Tzu Chi University, Hualien 970, Taiwan

**Keywords:** aryl hydrocarbon receptor, indoxyl sulfate, osteoclast, chronic kidney disease, NFATc1

## Abstract

Indoxyl sulfate (IS) is a chronic kidney disease (CKD)-specific renal osteodystrophy metabolite that affects the nuclear factor of activated T-cells, cytoplasmic 1 (NFATc1), a transcription factor promoting osteoclastogenesis. However, the mechanisms underlying the regulation of NFATc1 by IS remain unknown. It is intriguing that the Aryl hydrocarbon receptor (AhR) plays a key role in osteoclastogenesis, since IS is an endogenous AhR agonist. This study investigates the relationship between IS concentration and osteoclast differentiation in Raw 264.7 cells, and examines the effects of different IS concentrations on NFATc1 expression through AhR signaling. Our data suggest that both osteoclastogenesis and NFATc1 are affected by IS through AhR signaling in both dose- and time-dependent manners. Osteoclast differentiation increases with short-term, low-dose IS exposure and decreases with long-term, high-dose IS exposure. Different IS levels switch the role of AhR from that of a ligand-activated transcription factor to that of an E3 ubiquitin ligase. We found that the AhR nuclear translocator may play an important role in the regulation of these dual functions of AhR under IS treatment. Altogether, this study demonstrates that the IS/AhR/NFATc1 signaling axis plays a critical role in osteoclastogenesis, indicating a potential role of AhR in the pathology and abnormality of bone turnover in CKD patients.

## 1. Introduction

Uremic toxins have been associated with chronic kidney disease (CKD) progression, cardiovascular events, and bone mineral disease [1]. Indoxyl Sulfate (IS), a representative protein-binding uremic toxin, is produced by the intestinal bacteria as tryptophan is metabolized into indole [2]. IS enters renal tubular cells from the bloodstream via organic anion transporter (OAT) 1 and OAT3, and then drains into the renal tubules through OAT4 [3,4]. The deterioration in kidney function reduces the ability to remove IS and leads to increasing levels in the serum [5]; a high serum level of IS indicates severe CKD [6]. Fukagawa et al. have demonstrated that the accumulation of IS in the blood of CKD patients induces skeletal resistance to parathyroid hormone (PTH), inhibits intracellular cAMP production, decreases PTH receptor expression, and induces oxidative stress in osteoblasts [7]. Therefore, bone turnover rate tends to be low during the early stages of CKD [8].

Aryl hydrocarbon Receptor (AhR) has distinct physiological characteristics in vertebrates and invertebrates, and is essential for organ development. As a ligand-activated transcription factor, it is essential in regulating neural tube growth myelination [9], controlling drug metabolism [10], mediating inflammation [11] and xenobiotic metabolism, and in the tumorigenicity of dioxin carcinogens [12,13]. After binding to ligands, the complicated AhR compound translocates into the nucleus, dimerizes with the AhR nuclear translocator (ARNT), and acts as a transcriptional activator by binding with xenobiotic response element (XRE) sequences in promoter regions to regulate the expression of target genes such as cytochrome P450 family 1 subfamily A member 1 (CYP1A1) and CYP1B1 [14]. Ohtake et al. have reported that the unliganded AhR stays in the cytoplasm and correspondingly possesses ligand-dependent E3 ubiquitin ligase activity, resulting in proteasomal degradation and target protein ubiquitination [15]. The nuclear factor of activated T-cells, cytoplasmic 1 (NFATc1), one of AhR target proteins, plays an important role in osteoclast precursor differentiation [16] and is a major transcription factor for osteoclastogenesis [17].

It has been suggested that IS is a physiological activator of AhR or an AhR endogenous ligand [18], which crosses the bone cell membrane through OAT3 only [19] and affects AhR, controlling bone cellular processes [20]. However, in CKD mineral bone disease, IS-induced bone cell pathological conditions related to the AhR/NFATc1 signaling pathway have not been well characterized. This study aims to determine how IS regulates osteoclast AhR signaling and the mechanisms affecting NFATc1 expression after exposure to IS.

## 2. Results

### 2.1. Effects of IS on Osteoclast Differentiation

**Figure 1:**
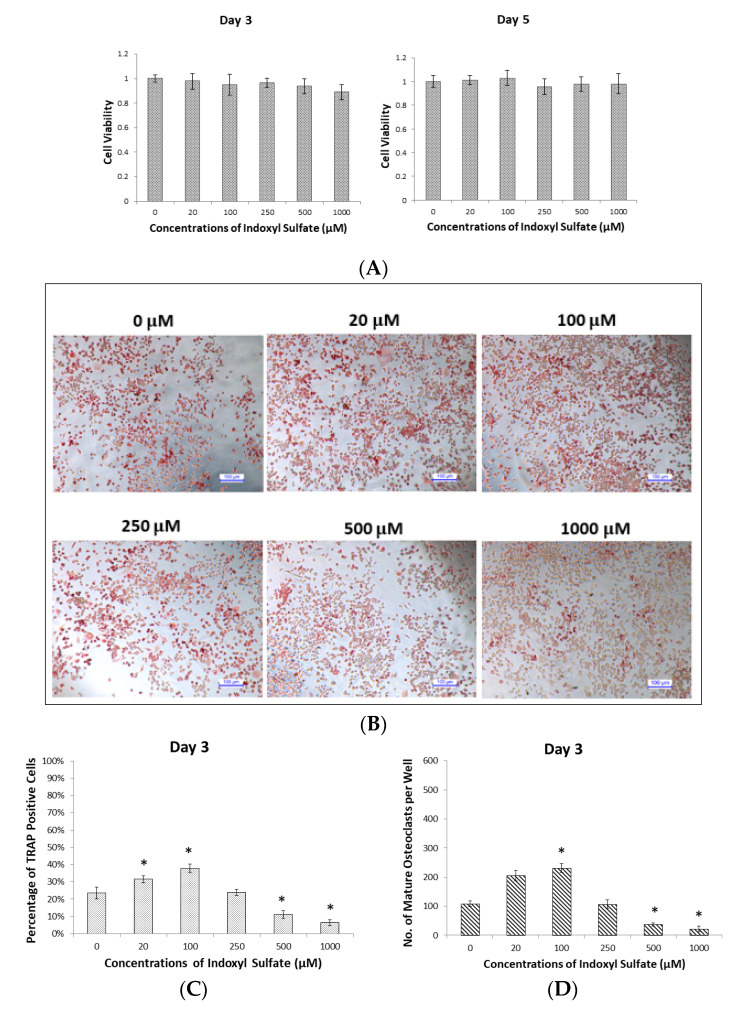

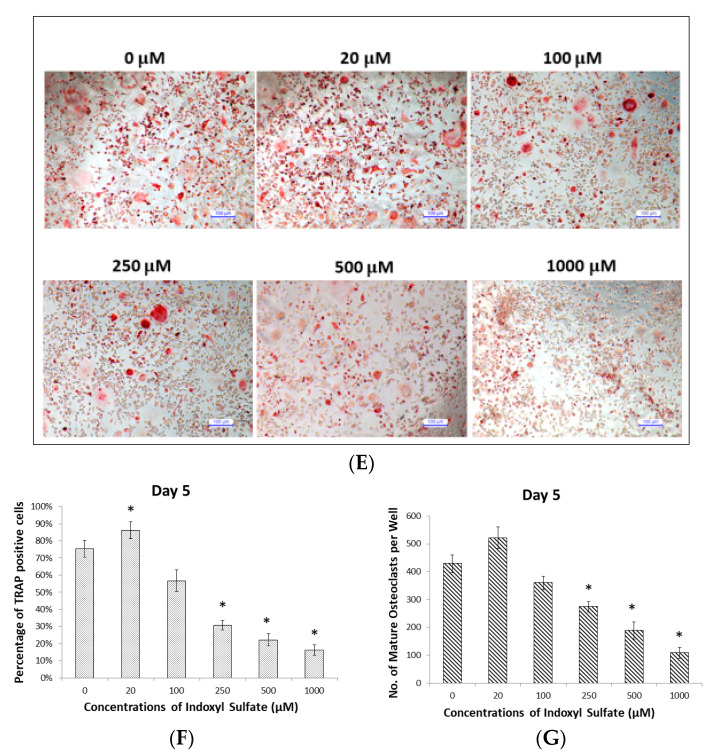
Dose- and time-dependent effects of indoxyl sulfate (IS) on osteoclast differentiation. Raw 264.7 cells with 50 ng/mL of sRANKL were cultured in IS at 0, 20, 100, 250, 500, and 1000 µM for 3 and 5 days. (**A**) The CCK-8 assay shows that cell viabilities are not significantly affected by low or high concentrations of IS on Days 3 or 5. Error bars represent mean ± SD (*n* = 8). (**B**) Representative images of TRAP stained cells on Day 3 after treatment with different concentrations of IS; TRAP-positive stained cells with one nucleus are recognizable as osteoclast precursor cells (undifferentiated osteoclast cells). (**C**) The average percentage of TRAP-positive cells in each group on Day 3; the highest percentage was at 100 µM IS. Error bars represent mean ± SD (*n* = 6). * *p* < 0.05 compared to the control group. (**D**) The average numbers of mature osteoclast cells (TRAP-positive stained cells with more than three nuclei) in each group on Day 3; the highest number was at 100 µM IS. Error bars represent mean ± SD (*n* = 6). * *p* < 0.05 compared to the control group. (**E**) Representative images of TRAP stained cells on Day 5 after treatment with different concentrations of IS; TRAP-positive stained with more than three nuclei and recognizable as mature osteoclast cells. (**F**) The average percentage of TRAP-positive cells in each group on Day 5; the highest percentage was at 20 µM IS. Error bars represent mean ± SD (*n* = 6). * *p* < 0.05 compared to the control group. (**G**) The average numbers of mature osteoclast cells in each group on Day 5; the highest number was at 20 µM IS. Error bars represent mean ± SD (*n* = 6). * *p* < 0.05 compared to the control group.

Raw 264.7 cells were cultured with 50 ng/mL of soluble Receptor Activator of Nuclear Factor-κB Ligand (sRANKL) in IS at 0, 20, 100, 250, 500, and 1000 µM for cell viability measurements on Day 3 (osteoclast precursor stage) and Day 5 (osteoclast mature stage). The cell counting kit-8 (CCK-8) assay revealed that no concentration of IS significantly affected cell viability at the osteoclast precursor or osteoclast mature stage (Figure 1A).

Tartrate-resistant acid phosphatase (TRAP) was used as a marker of osteoclast differentiation [21]. Raw 264.7 cells cultured with different concentrations of IS were examined with a TRAP staining kit on Days 3 and 5 to evaluate the state of osteoclastogenesis (Figure 1B,C). On Day 3 (osteoclast precursor stage), most of TRAP-positive stained cells were precursor osteoclast cells (with one nucleus) or undifferentiated osteoclasts, and there were few mature osteoclast cells (TRAP-positive staining of more than three nuclei) (Figure 1B). IS concentrations of 20 and 100 µM increased the percentage of TRAP-positive cells (Figure 1C), implying that 3 day cultivation with lower concentrations (20 and 100 µM) promotes osteoclast precursor cells growth, but that high concentrations (500 and 1000 µM) block it. In addition, the numbers of mature osteoclast cells showed (Figure 1D) a similar variation to the percentage of TRAP-positive cells.

On Day 5 (osteoclast mature stage), the percentage of TRAP-positive cells and numbers of mature osteoclast cells were higher than on Day 3 in all groups. The group treated with 20 µM IS had the highest percentage of TRAP-positive cells (Figure 1F) and number of mature osteoclast cells (Figure 1G). Furthermore, the percentage of TRAP-positive cells and the numbers of mature osteoclast cells decreased in dose-dependent manner as the IS concentration increased above 100 µM. This phenomenon suggests that after 5 days of IS incubation, 20 µM IS stimulated, but higher concentrations (>100 µM) inhibited, mature osteoclast differentiation.

### 2.2. IS Activates AhR Transcription Factor Signaling in Osteoclasts

**Figure 2:**
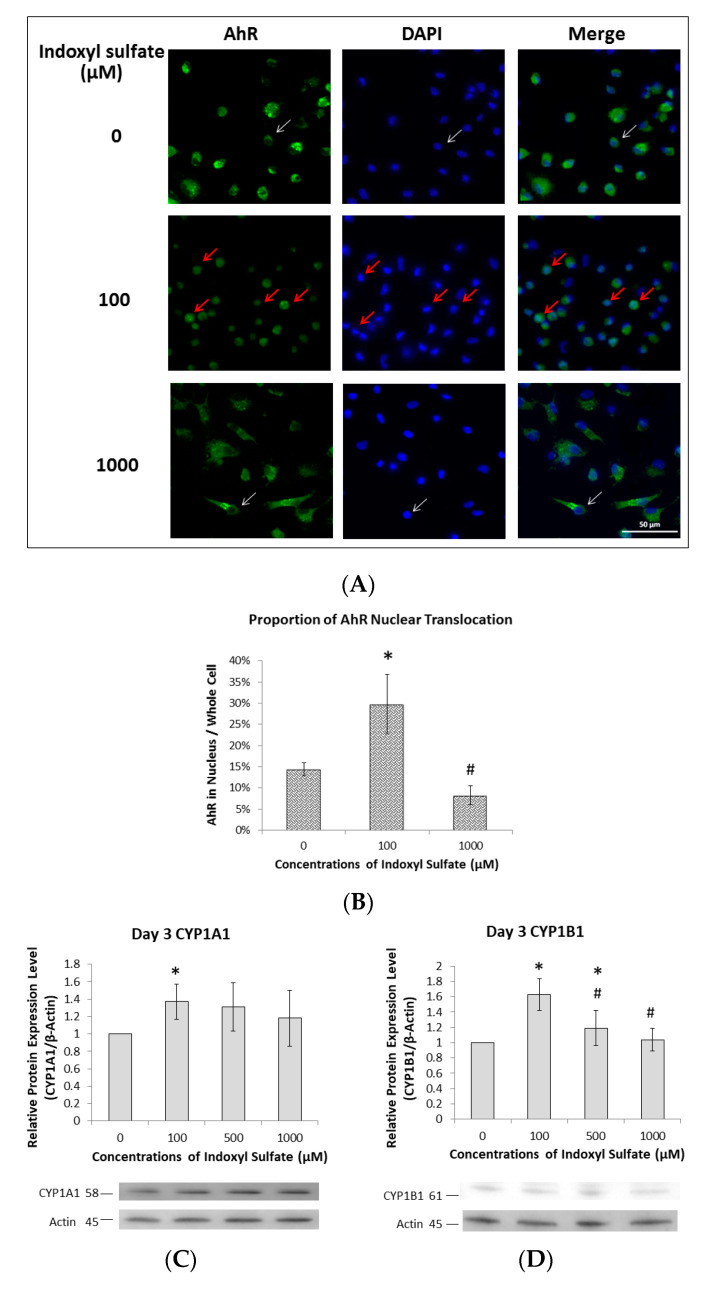

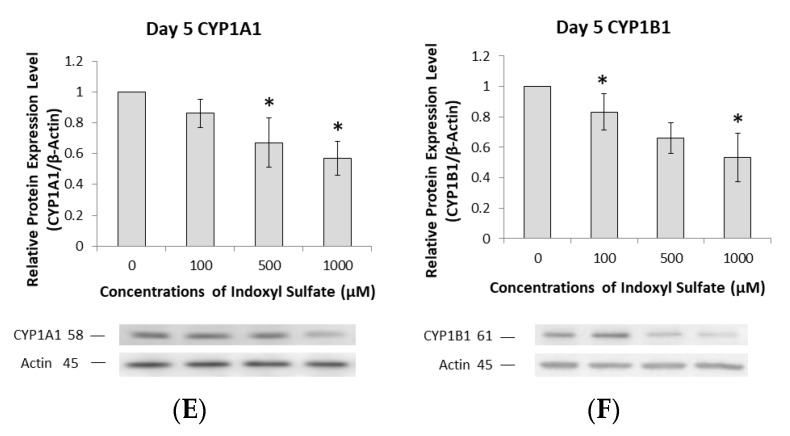
Effects of low IS concentrations on AhR transcription, nuclear translocation, and CYP enzyme production in osteoclasts. Raw 264.7 cells with 50 ng/mL of sRANKL were cultured in IS at 0, 100, 500, and 1000 µM for 3 and 5 days. (**A**) Low IS concentrations increase AhR nuclear translocation on Day 3. Immunofluorescence with an anti-AhR antibody revealed that the most AhR localization (green) occurs in the cytoplasm with 0 and 1000 µM IS. White arrows indicate that AhR is present in the cytoplasm and not the nucleus. Red arrows indicate that AhR is present in both the cytoplasm and nucleus (at 100 µM IS). A representative example from three independent experiments is shown. (**B**) Average ratio of AhR nuclear translocation in immunofluorescence images for each group on Day 3; the highest percentage was at 100 μM IS. Error bars represent mean ± SD (*n* = 5). * *p* < 0.05 compared to control group. # *p* < 0.05 compared to 100 μM IS. (**C**,**D**): CYP1A1 and CYP1B1 expression, respectively, at different IS concentrations on Day 3. Western blot analysis showed that low IS concentrations (100 µM) increased CYP1A1 and CYP1B1 expression, while high IS concentrations (>500 µM) inhibited CYP1A1 and CYP1B1 expression. Error bars represent mean ± SD (*n* = 3). * *p* < 0.05 compared to the control group. # *p* < 0.05 compared to 100 µM IS. (**E**) and (**F**): CYP1A1 and CYP1B1 expression, respectively, at different IS concentrations on Day 5. Western blot analysis showed that IS treatment inhibited CYP1A1 and CYP1B1 expression in a dose-dependent manner. Error bars represent mean ± SD (*n* = 3). * *p* < 0.05 compared to the control group.

After culturing Raw 264.7 cells with or without IS, the immunofluorescence of osteoclast precursors was observed using an anti-AhR antibody and DAPI on Day 3. Without IS, AhR fluorescence was observed in the cytoplasm but not in the nucleus. This could indicate that without IS, unliganded AhR remains in the cytoplasm and forms a compound with a heat shock protein 90 (HSP90) dimer, p23, AhR-interacting protein (AIP), and protein kinase SRC [22]. However, when the cells were cultured with 100 µM IS, AhR fluorescence appeared in both the cytoplasm and nucleus, indicating AhR nuclear translocation. When they were cultured with 1000 µM IS, AhR fluorescence was observed in the cytoplasm but not the nucleus, indicating that this concentration inhibited nuclear translocation (Figure 2A,B).

Nuclear ligand-activated AhR can upregulate prototypic target genes such as CYP1A1 and CYP1B1. We observed maximum CYP family expression with 100 µM IS on Day 3 (Figure 2C,D). Furthermore, CYP family expression was reduced as IS exposure increased (Figure 2E,F), suggesting that high concentrations of IS prevent AhR translocation.

### 2.3. AhR Pathway Mediates IS Regulation of NFATc1

**Figure 3:**
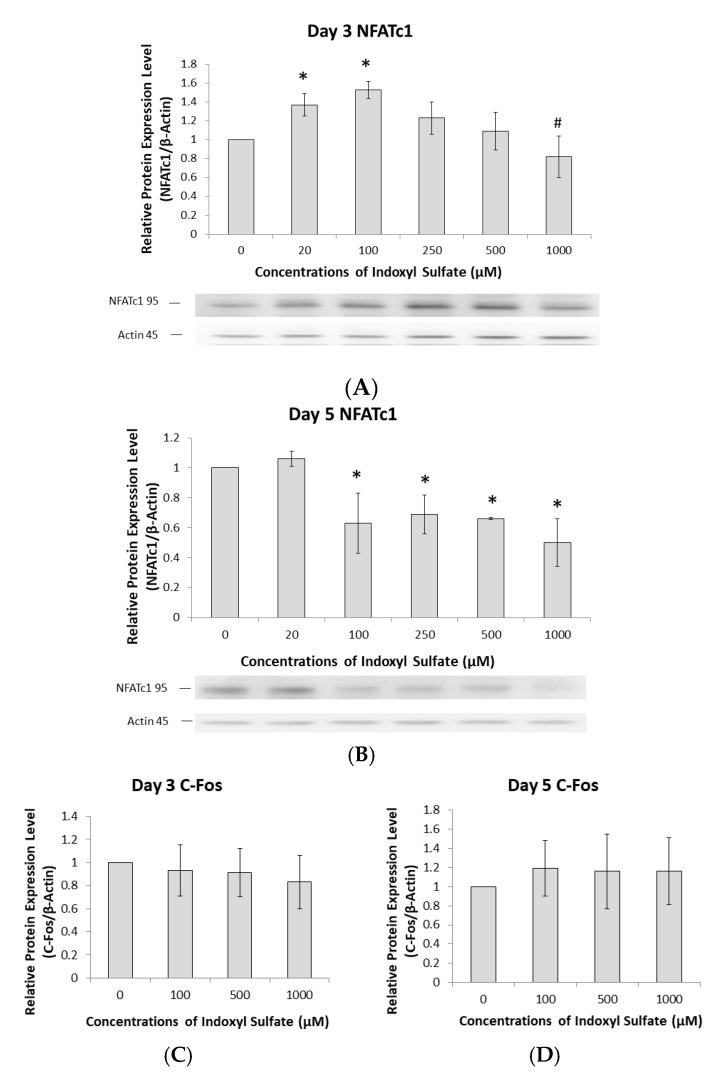
The AhR pathway mediates IS regulation of NFATc1 but not c-Fos. Raw 264.7 cells with 50 ng/mL of sRANKL were cultured in IS at 0, 100, 500, and 1000 µM for 3 and 5 days. (**A**) NFATc1 expression in osteoclast precursors on Day 3 depends on the IS concentration. Western blot analysis showed that <100 µM IS increased NFATc1 expression, but >250 µM inhibited it in a dose-dependent manner. Error bars represent mean ± SD (*n* = 3). * *p* < 0.05 compared to the control group. # *p* < 0.05 compared to 100 µM IS. (**B**) NFATc1 expression in mature osteoclasts on Day 5 depends on the IS concentration. Western blot analysis showed that 20 µM IS increased NFATc1 expression, but >100 µM decreased it. Error bars represent mean ± SD (*n* = 3). * *p* < 0.05 compared to the control group. (**C**,**D**) c-Fos expression in osteoclasts at different IS concentrations on Day 3 and 5. Western blot analysis showed no significant relationship between c-Fos expression and IS concentrations. Error bars represent mean ± SD (*n* = 4).

NFATc1 plays a key role in the transcriptional regulation of osteoclast differentiation [16]. To understand how IS regulates NFATc1, the correlation of NFATc1 expression with IS treatment was examined. Raw 264.7 cells were exposed to different IS concentrations for 3 days. Treatment with 20 and 100 µM IS increased NFATc1 expression (Figure 3A), but expression was dose-dependently decreased by 500 and 1000 µM (Figure 3A). On Day 5, NFATc1 expression was attenuated in mature osteoclasts when the IS concentration was greater than 100 µM (Figure 3B). Nevertheless, our data suggest that IS can regulate the NFATc1 pathway to influence osteoclast differentiation. However, C-Fos protein expression was not dependent on the IS concentration at either Day 3 or 5 (Figure 3C,D).

### 2.4. Regulation of NFATc1 Expression by AhR Antagonism

**Figure 4:**
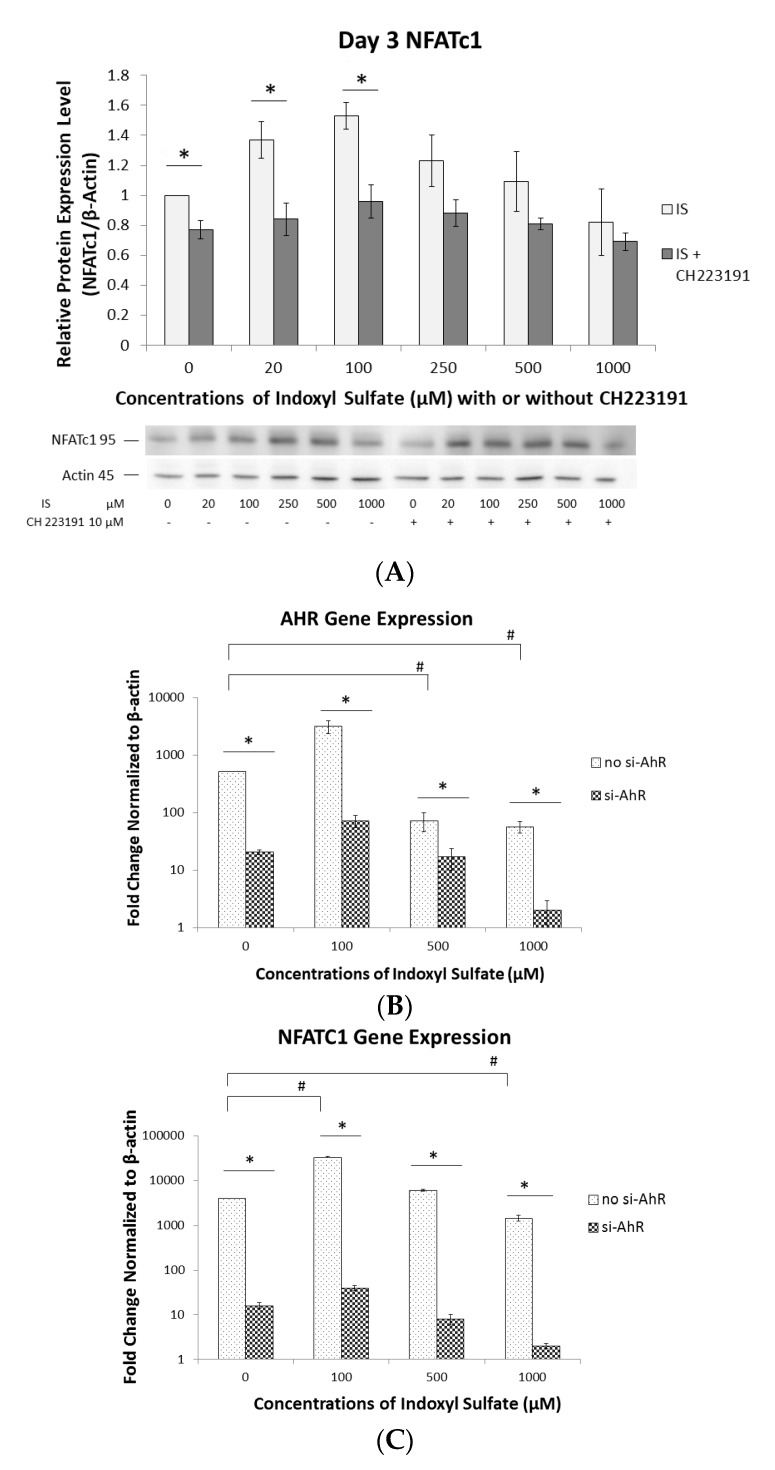
Effect of AhR antagonism on NFATc1 expression. (**A**) Raw 264.7 cells with 50 ng/mL of sRANKL were cultured in IS at 0, 100, 500, and 1000 µM for 3 or 5 days. NFATc1 expression was inhibited by CH223191 on Day 3. Western blot analysis showed that NFATc1 expression was significantly inhibited by CH223191 at <100 µM IS. Error bars represent mean ± SD (*n* = 3). * *p* < 0.05 compared to with or without CH223191 at individual IS concentrations. (**B**) Effect of AHR knockdown on AHR gene expression. Raw 264.7 cells with or without transfected AHR siRNA (si-AHR) were cultured for 24 h and then treated with different IS concentrations for another 48 h. The fold changes in gene expression levels were determined by real-time quantitative polymerase chain reaction (qRT-PCR) and calculated relative to the control group, normalized to actin. The expression of the AHR gene without transfected si-AHR was significantly lower in the 500 and 1000 µM IS treatment groups than in the 0 µM group. Its expression in the si-AHR groups was significantly lower than in the non-si-AHR groups. Error bars represent mean ± SD (*n* = 3). * *p* < 0.05 compared with and without transfected si-AHR. # *p* < 0.05 compared with 0 µM IS in non-si-RNA groups. (**C**) Effect of AHR knockdown on NFATC1 gene expression levels. Raw 264.7 cells with or without transfected AHR siRNA (si-AHR) were cultured for 24 h and treated with different IS concentrations for another 48 h. The fold changes in gene expression levels were determined by qRT-PCR and calculated relative to the control group, normalized to actin. NFATC1 expression without transfected si-AHR was significantly lower in the 1000 µM IS treatment group than in the 0 µM group. NFATC1 expression without transfected si-AHR was significantly higher in the 100 µM IS treatment group than in the 0 µM group. NFATC1 expression was significantly lower in the si-AHR groups than in the non-si-AHR groups. Error bars represent mean ± SD (*n* = 3). * *p* < 0.05 compared with and without transfected si-AHR. # *p* < 0.05 compared with 0 µM IS in non-si-RNA groups.

To verify that AhR can also regulate NFATc1, we used two models: (1) an AhR antagonist, and (2) an AHR small interfering RNA (si-AHR) transfection. To determine protein levels, 10 µM of CH223191 (an AhR antagonist, which potently blocks ligand-induced AhR-dependent nuclear translocation [23]) was added to various concentrations of IS. NFATc1 expression was significantly attenuated by CH223191 on Day 3 with <100 µM IS (Figure 4A). To determine the expression of the AHR and NFATC1 genes, Raw 264.7 cells were transfected with or without si-AHR for 24 h and treated with different concentrations of IS for another 48 h. Without si-AHR transfection, the gene expression of both AHR and NFATC1 peaked at 100 µM IS and decreased at 500 and 1000 µM (Figure 4B,C). By contrast, AHR and NFATC1 expression was significantly lower in the si-AHR transfected groups than in those without si-AHR (Figure 4B,C). These results demonstrated that in the si-AHR transfected group, AHR gene expression is suppressed several hundred-fold with low or high concentrations of IS. Consequently, the expression of NFATC1 is suppressed several thousand-fold with low or in high concentrations of IS. This suggests that NFATC1 is downstream of AHR and that IS can regulate NFATC1 expression via AHR signaling.

### 2.5. NFATc1 Ubiquitination and ARNT Expression Depend on IS Concentration

Apart from being a transcription factor, AhR also acts as an E3 ubiquitin ligase to target certain proteins for proteasomal degradation in a ligand-dependent manner. Our aim was to determine the exact mechanism by which ARNT regulates this dual function of AhR under IS exposure.

**Figure 5:**
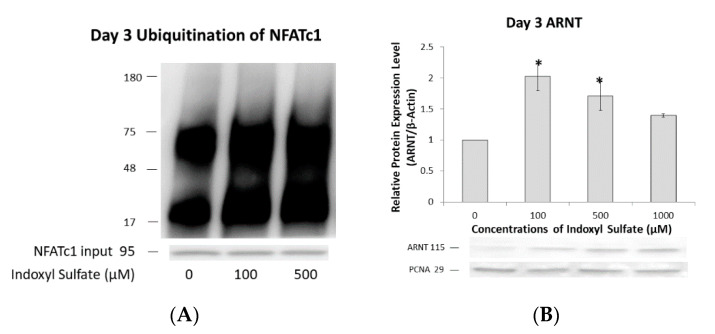
IS dose-dependent NFATc1 ubiquitination and ARNT expression. Raw 264.7 cells with 50 ng/mL of sRANKL were cultured in IS at 0, 100, 500, and 1000 µM for 3 days. (**A**) NFATc1 ubiquitination on Day 3 depended on the IS level. NFATc1 ubiquitination at lower IS levels (100 µM) is low, compared to that at higher IS levels (1000 µM). Immunoprecipitated NFATc1 is shown as an input. A representative example of three independent experiments is shown. (**B**) ARNT expression on Day 3 depended on the IS level. Western blot analysis showed that low IS levels (100 µM) increased nuclear ARNT expression, while high levels (>500 µM) inhibited it. Error bars represent mean ± SD (*n* = 3). * *p* < 0.05 compared to the control group.

On Day 3, osteoclast precursors were examined for NFATc1 ubiquitination. At a low IS concentration (100 µM), AhR mainly functioned as a ligand-activated transcription factor for NFATc1, with a decrease in NFATc1 ubiquitination. By contrast, proteasomal degradation and NFATc1 ubiquitination increased at high IS concentrations (1000 µM); that is, the E3 ubiquitin ligase activity of AhR increased (Figure 5A). Additionally, ARNT expression was increased at low IS concentrations (100 µM) but decreased at high concentrations (1000 µM; Figure 5B). This correlation between ARNT and NFATc1 ubiquitination indicates that different IS concentrations can control ARNT to act as a molecular switch determining whether AhR serves as a ligand-activated transcription factor or an E3 ubiquitin ligase. Therefore, the IS concentration affects NFATc1 ubiquitination and proteasomal degradation.

**Figure 6:**
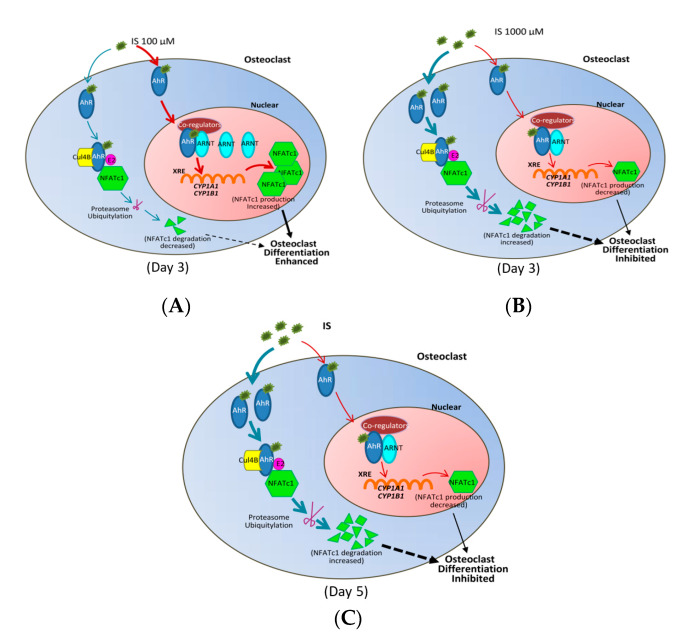
ARNT is an important modulator of AhR’s dual functions under IS treatment. (**A**) ARNT switches AhR from a ligand-activated transcription factor to an E3 ubiquitin ligase under IS treatment on Day 3. At 100 µM IS, ARNT is available and AhR functions as a ligand-activated transcription factor, increasing CYP1A1 and CYP1B1 expression to promote NFATc1 expression, thereby increasing osteoclast precursor differentiation. The red pathway represents the production of NFATc1, and the blue represents the degradation of NFATc1. (**B**) On Day 3 at 1000 µM IS, ARNT is inaccessible and AhR functions as an E3 ubiquitin ligase, leading to the proteasomic degradation of NFATc1, thereby inhibiting osteoclast precursor differentiation. The red pathway represents the production of NFATc1, and the blue pathway represents the degradation of NFATc1. (**C**) ARNT controls AhR to act as an E3 ubiquitin ligase under IS treatment on Day 5. As ARNT expression is inhibited, the transcriptional activity of AhR is blocked, while its E3 ubiquitin ligase function is enhanced, thereby increasing NFATc1 ubiquitination and inhibiting osteoclast differentiation. The red pathway represents the production of NFATc1, and the blue, the degradation of NFATc1.

In summary, as schematically shown in Figure 6, the IS concentration can influence NFATc1 expression through ARNT expression and AhR signaling.

## 3. Discussion

IS accumulates in the blood of CKD patients and contributes to the commonly observed deterioration of bone metabolism. In this investigation, we primarily found that IS can influence osteoclastogensis in a dose- and time-dependent manner. With short-term IS exposure (3 days), a low concentration of IS promoted osteoclast precursor differentiation. However, longer exposure (5 days) resulted in the attenuation of mature osteoclast differentiation. This longer IS-exposure situation is similar to the CKD scenario, in which osteoclast differentiation might be suppressed.

We further proved that IS regulates osteoclast differentiation via AhR binding, thereby influencing NFATc1 expression. Under long-term IS exposure, NFATc1 expression is decreased by IS in a dose-dependent manner. Furthermore, we observed that IS could regulate ARNT expression to regulate the dual functions of AhR. Short-term and low-dose IS exposure increases nuclear ARNT expression, facilitating the formation of the IS/AhR/NFATc1 complex in the nucleus. However, exposure to high IS concentrations decreases ARNT expression, inhibiting the entry of IS into the nucleus and increasing AhR E3 ligase activity. This is the first study that has determined the effects of different IS doses and times on osteoclast differentiation.

Mozar et al. reported that IS dose-dependently inhibited osteoclast differentiation and functioning via the ERK1/2, p38, JNK, and Akt pathways after culturing with >200 µM of IS for 5 days [24]. Watanabe et al. found that different IS concentrations (30, 100, and 300 µM) blocked Raw 264.7 cells, osteoclast precursors, and bone marrow-derived macrophages development, as well as inhibiting RANKL-induced differentiation into mature osteoclasts, after culturing for 5 days [19]. In the present study, we also observed that IS suppressed osteoclast differentiation after long-term exposure, but that IS enhanced osteoclast development after short-term, low-dose exposure, which has not previously been reported.

AhR signaling also plays an important role in osteoclastogenesis [25]. Wejheden et al. reported that low levels of constitutively active AhR increased bone resorption in female mice [26]. Iqbal et al. reported that smoke toxins, such as benzo[a]pyrene (BaP) and 2,3,7,8-tetrachlorodibenzo-p-dioxin (TCDD), interact with AhR as exogenous ligands to induce osteoclast bone resorption [12]. Yu et al. demonstrated that bone mass increased with decreasing bone resorption in AhR knockout mice [27], and that 3-methylcholanthrene, an AhR agonist and a carcinogenic polycyclic aromatic hydrocarbon, cannot induce bone loss if osteoclasts are deficient in AhR [28]. However, Korkalainen et al. reported that TCDD inhibits osteoclastogenesis by decreasing TRAP-positive cells and bone resorption areas [29]. These contrasting results illustrate that AhR, when binding with different ligands, has different functions that are affected by the nature of the binding ligand, binding time, and specific pathways of distinct ligands [30].

IS activates AhR signaling in the following manner. Short-term, low-dose IS exposure promotes the nuclear translocation and transcriptional activity of AhR and upregulates the expression of prototypic target genes (*CYP1A1*, *CYP1B1*). Then, ligand-bound AhR is degraded and exported from the nucleus [31,32]. We determined that NFATc1 expression depends on the concentration and time of IS treatment, consistent with the performance of TRAP stain-positive osteoclasts induced by IS. C-Fos is another important transcription factor in osteoclastogenesis [33]; c-Fos-deficient mice have been shown to develop osteopetrosis due to inadequate osteoclast differentiation [34]. Izawa et al. reported that 3-day exposure to BaP (0.5 µM) stimulated higher c-Fos expression in a wild-type osteoclastogenesis model, but not in an AhR knockout model [25]. This result was contradictory to our results (c-Fos expression was not related to IS levels). Therefore, we hypothesize that the IS/AhR/NFATc1 pathway regulates osteoclast precursor differentiation.

AhR has recently been found to have dual functions [15]. Besides acting as a transcription factor, it possesses an intrinsic E3 ubiquitin ligase function, which induces the proteasomal degradation and ubiquitination of target proteins. In the present study, at the osteoclast precursor stage, AhR functioned as a transcription factor, and NFATc1 expression was enhanced by a low IS concentration. Short-term, low-dose IS exposure increased NFATc1 expression, not only by increasing AhR transcriptional activity to enhance NFATc1 production, but also by decreasing AhR E3 ligase activity to attenuate NFATc1 ubiquitination. However, higher IS concentrations stimulated the AhR E3 ubiquitin ligase pathway, enhancing NFATc1 ubiquitination and diminishing NFATc1 expression.

We found that AhR antagonists acted to counteract the effects of IS treatment to establish the IS/AhR/NFATc1 pathway. The AhR antagonist CH223191 prevented the AhR transcription factor’s function on osteoclast precursors at low IS concentrations, thus inhibiting NFATc1 expression. We performed qRT-PCR to inspect AHR and NFATC1 gene expression and found that both genes were identically reduced when IS-treated Raw 264.7 cells were transfected with si-AHR. Hence, we confirmed that AhR is an upstream activator of NFATc1. However, our results were different from those of a study conducted by Parsa et al., who emphasized that AhR expression depends on the activation of NFATc1, and that cyclosporin A (CsA)—a NFATc1 inhibitor—remarkably inhibits AhR expression in lung tissue affected by BaP [35]. Furthermore, we noticed that the si-AHR was more efficient than CH223191 in blocking AhR/NFATc1 signaling, suggesting that other cellular proteins probably interfered with the inhibitory effects of the AhR antagonist.

When ligand-activated AhR binds to ARNT (also known as HIF1β) in the nucleus, the AhR–ARNT complex is recruited to XREs [36]. In addition, the binding of ligands to AhR results in the formation of the AhR–cullin 4B ubiquitin ligase complex (CuL4B), which degrades other transcription factors such as NFATc1 and c-Fos [37]. This AhR E3 ligase function competes with its transcription factor activity, which is dependent on ARNT [38]. Luecke-Johansson et al. have suggested that ARNT is the essential regulator of the dual functions of AhR. They found that a lack of ARNT seriously hampered the transcriptional activation function of AhR but augmented its E3 ubiquitin ligase function [38]. Our study demonstrated that ARNT expression is upregulated in the osteoclast precursor stage under low IS concentrations, and that the AhR transcriptional function is activated to increase NFATc1 production. By contrast, high IS concentrations suppress ARNT expression to enhance NFATc1 ubiquitination. Therefore, we determined that the IS concentration can control ARNT to function as a molecular switch, determining whether AhR serves as a ligand-activated transcription factor for NFATc1 expression in the nucleus or a part of the ubiquitin ligase complex for NFATc1 proteasomal degradation in the cytoplasm.

We conclude that osteoclast differentiation can be suppressed by high concentration and long-term IS exposure. To date, there is no treatment available to rescue osteoclasts in CKD patients. Although uremic toxin elimination may be an option for correcting abnormal osteoclast development, we cannot provide novel evidence showing that AhR, in cooperation with NFATc1, plays an important role in controlling osteoclast differentiation. The IS/AhR/NFATc1 pathway prevents the CKD-associated deterioration of bone metabolism, and therefore, AhR antagonists may serve as novel drugs for renal osteodystrophy. Although RAW 264.7 cells have been used as immortalized murine macrophage cells for more than four decades [36], we can still consider, as a limitation of the study, the need to profile other primary cells to verify this concept. There may be different metabolic pathways among human cells, and this could be another interesting issue for investigation. Future studies exploring the therapeutic advantages of targeting AhR to improve CKD-related bone disease and to better understand the contributions of AhR to osteoclastogenesis are warranted.

## 4. Materials and Methods

### 4.1. Raw 264.7 Cell Culturing and IS Treatment

The mouse monocyte cell line Raw 264.7 was obtained from the Bioresource Collection and Research Center (Hsinchu, Taiwan). The culture medium included 90% α-MEM (Gibco, Las Vegas, NV, USA), 10% Fetal Bovine Serum (Gibco, Las Vegas, NV, USA), 1% Antibiotic-Antimycotic (100X) (Gibco, USA), and 50 ng/mL of soluble Receptor Activator of Nuclear Factor-κB Ligand (sRANKL) (PeproTech, Rehovet, Israel). The medium was replaced on Day 3, and the cells were incubated for 3–5 days in a 5% CO_2_ atmosphere at 37 °C. Usually, after 3 days of culturing with sRANKL, raw cells become osteoclast precursor cells or undifferentiated osteoclasts (with one nucleus). To obtain cells considered as mature osteoclast cells (with more than three nuclei), raw cells were cultured in the medium for 5 days.

IS (Sigma-Aldrich, St. Louis, MO, USA) was dissolved in DMSO (Sigma-Aldrich, St. Louis, MO, USA), according to the manufacturer’s instructions. Vanholder et al. observed that the normal concentration in a healthy population is 2 μM, the mean/median uremic concentration for a uremic population is 211 μM (53 ± 91.5 mg/L) in CKD patients, and the maximal uremic concentration for the uremic population is 940 μM (236 mg/L) [39]. In this study, Raw cells were treated with different concentrations of IS to mimic its effects on osteoclasts at various stages in CKD patients. Concentrations of IS ≤ 100 μM were used as low doses and those ≥500 µM as high doses.

The working concentration of CH223191 (Sigma-Aldrich, St. Louis, MO, USA) was 10 µM. The CH223191 was pretreated for 4 h before IS was added to the medium.

### 4.2. CCK-8 Assay and TRAP Staining Kit

To measure cell viabilities, Raw 264.7 cells (5 × 10^4^ cells/well) were seeded in 96-well plates with low to high concentrations of IS for 3 and 5 days respectively. The Cell Counting Kit-8 (CCK-8) assay kit (Dojindo Molecular Technologies, Inc., Rockville, MD, USA) was used to determine absolute values through the Cell Proliferation Assay according to manufacturer’s instructions. The CCK-8 contained the 2-(2-methoxy-4-nitrophenyl)-3-(4-nitrophenyl)-5-(2,4-disulfophenyl)- 2H-tetrazolium monosodium salt, WST-8 [40]. WST-8 reacts with the electron carrier 1-methoxy PMS to form a water-soluble, yellow-colored formazan dye, detectable at a wavelength of 450 nm.

The Raw 264.7 cells (8 × 10^5^ cells/well) were seeded in 6-well plates for observation on Day 3 or 5. The cells were then fixed with 4% paraformaldehyde for 15 min and incubated with the TRAP staining kit (Sigma-Aldrich, St. Louis, MO, USA). Staining images were observed under a microscope (Carl Zeiss, Germany) with random fields in each group to calculate the percentages of TRAP-positive stained cells and the numbers of mature osteoclast cells in each well on Days 3 and 5.

### 4.3. Immunofluorescence

To analyze the AhR distribution, 1 × 10^6^ Raw 264.7 cells were cultured in 3.5 cm dishes with cover slides. After 3 days, the cells were fixed with 4% paraformaldehyde for 15 min and incubated overnight with 0.2% AhR primary antibody (Thermo Fisher Scientific, Waltham, MA, USA) at 4 °C, and then subsequently with the 0.5% fluorescein isothiocyanate AffiniPure donkey anti-rabbit immunoglobulin G (IgG; H+L) secondary antibody (Jackson ImmunoResearch Laboratories, Inc., West Grove, PA, USA) for 90 min at room temperature. Finally, the cells were stained with DAPI (Southern Biotech, Birmingham, AL, USA) on glass slides and examined under a Zeiss LSM 510 Confocus microscope (Carl Zeiss AG, Oberkochen, Germany). Images were obtained using a digital AxioCam HRm camera system (Carl Zeiss). The Axio Vision Measurement Program for the AxioCam MRc (Carl Zeiss) was used to quantify AhR nuclear translocation at different concentrations of IS.

### 4.4. Western Immunoblotting and Ubiquitination

Raw 264.7 cells were lysed with RIPA buffer (Bio Basic Inc., Toronto, Canada) containing a full range-protease inhibitor cocktail (BIONOVAS, Toronto, Canada). The nuclear extract was isolated from the cells using the Nuclear/Cytosol Fractionation Kit (BioVision, Inc., Milpitas, CA, USA). Then, 50 mg of protein was loaded into each well of, and separated in, an SDS–PAGE gel, and subsequently transferred to a polyvinylidene fluoride membrane using a semidry transfer apparatus (Bio-Rad Laboratories, Inc., Hercules, CA, USA) and blocked for 1 h in 5% milk in Tris-buffered saline and 0.1% Tween 20. Primary antibody was added and the mixture was incubated overnight at 4 °C, followed by secondary antibody incubation for 1 h before adding the Western Bright Quantum horseradish peroxidase substrate (Advansta, Inc., San Jose, CA, USA). Images were taken using a chemiluminescence imager (Syngene, UK) with an ECL substrate (GE Healthcare, Chicago, IL, USA). The primary antibodies used were against AhR, cytochrome P450 family 1 subfamily A member 1 (CYP1A1; GeneTex, Hsinchu City, Taiwan), CYP1B1 (GeneTex, Hsinchu City, Taiwan), NFATc1 (Santa Cruz Biotechnology, Dallas, TX, USA), c-Fos (Abcam, Cambridge, UK), ARNT (Abnova, Taipei, Taiwan), Actin (Proteintech, Rosemont, IL, USA), and PCNA (GeneTex, Hsinchu City, Taiwan).

NFATC1 ubiquitination was detected with an immunoprecipitation assay. Before lysis, Raw 264.7 cells were treated with 10 µM of MG132 (Cayman Chemical Company, Ann Arbor, MI, USA) for 4 h. Cell extracts were incubated with the NFATC1 primary antibody for immunoprecipitation overnight at 4 °C. On the following day, cell lysates were incubated with Protein A/G agarose (Santa Cruz Biotechnology) beads for 1.5 h at room temperature and washed five times with RIPA buffer for ubiquitin antibody immunoblotting analysis.

### 4.5. qRT-PCR and siRNA Transfection

Total cellular RNA was isolated from the cells using the Tools sharp RNA Extractor kit (Tools, Taiwan) and quantified using a Nanodrop 2000 (Thermo Fisher, Waltham, MA, USA). The DNase I Amplification Kit (Invitrogen, USA) was used to digest genomic DNA in 2 μg of RNA solution, and then the iScript cDNA Synthesis Kit (Bio-Rad, Hercules, CA, USA) was used for reverse transcription. The reaction mixture was incubated for 10 min at 25 °C, and then for 20 min at 46 °C for reverse transcription, followed by incubation for 1 min at 95 °C to inactivate the reverse transcriptase. A real-time quantitative polymerase chain reaction (RT-qPCR) protocol was developed to measure the expression levels of the studied genes in the osteoclasts. Table 1 lists the primers used for qRT-PCR. All PCR primers were synthesized by Genomics (Taiwan). The RT reactions were run for 40 cycles in 96-well reaction plates using the LightCycler^®^ 480 Instrument II (Roche Molecular Systems, Inc., Pleasanton, CA, USA). qPCR was performed using the cycling program of the Simply Green qPCR Master Mix detection protocol. Following amplification, cooling was performed at 40 °C. All runs were completed with melt curve analysis to confirm the amplification specificity and a lack of primer dimers.

Small interfering RNA (siRNA) oligonucleotides directed against mouse AhR (si-AhR; Si Genome SMART Pool Mouse Ahr) were obtained from Perbio Science, France. The siRNA transfections were performed in 24-well culture plates with Raw 264.7 cells in the presence of DharmaFECT Transfection Reagent 1 (Horizon Discovery, Waterbeach, UK). siRNA (5 nmol) and 2.0 μL of TransFectin reagent were added to each well, adding α-MEM to a final volume of 500 μL, and the cells were cultured for 24 h. After siRNA transfection, the transfected cells were cultured in antibiotic-free α-MEM with 50 ng/mL of sRANK Ligand and 10% FBS for 48 h.

### 4.6. Statistical Analysis

Data are presented as mean ± standard deviation (SD), and at least three independent experiments were performed per condition. The data were analyzed using the SAS 9.0 software (SAS Institute Inc., Cary, NC, USA), and *p* < 0.05 was considered to be statistically significant.

## Figures and Tables

**Table 1:** List of primer sequences used for qRT-PCR analysis in this study.

Gene	Forward Primer	Reverse Primer
AhR	5′-TTCTTAGGCTCAGCGTCAGCTA-3′	5′-GCAAATCCTGCCAGTCTCTGAT-3′
FATc1	5′-GACTTCGATTTCCTCTTCGAGTTC-3′	5′-CTCGATTCTCGGACTCTCCAG-3′
β-Actin	5′-CCTCTATGCCAACACGTGC-3′	5′-CCTGCTTGCTGATCCACATC-3′

## Abbreviations

AhR	Aryl hydrocarbon Receptor
AIP	AhR-interacting protein
ARNT	Aryl hydrocarbon Receptor Nuclear Translocator
BaP	Benzo[a]pyrene
CCK-8 assay	Cell counting kit-8 assay
CKD	Chronic kidney disease
CsA	Cyclosporine A
CuL4B	Cullin 4B ubiquitin ligase complex
CYP1A1	Cytochrome P450 family 1 subfamily A member 1
CYP1B1	Cytochrome P450 family 1 subfamily B member 1
HSP90	Heat shock protein 90
IS	Indoxyl sulfate
NFATc1	Nuclear factor of Activated activated T-cells, cytoplasmic 1
OAT	Organic anion transporter
qRT-PCR	Real-time quantitative polymerase chain reaction
PTH	Parathyroid hormone
si-AHR	AHR small interfering RNA
si-RNA	Small interfering RNA
SD	Standard Deviation
sRANKL	Soluble Receptor Activator of Nuclear Factor-κB Ligand
TCDD	2,3,7,8-tetrachlorodibenzo-p-dioxin
TRAP	Tartrate-resistant acid phosphatase
XRE	Xenobiotic response element
